# How the Nursing Practice Environment Influences Retention and Turnover Intention: An Umbrella Review Protocol

**DOI:** 10.3390/nursrep14040235

**Published:** 2024-10-29

**Authors:** Ana Rita Figueiredo, Filomena Gaspar, Cristina Baixinho, Pedro Lucas

**Affiliations:** Nursing Research, Innovation and Development Centre of Lisbon (CIDNUR), Nursing School of Lisbon (ESEL), 1600-190 Lisbon, Portugal; mfgaspar@esel.pt (F.G.); crbaixinho@esel.pt (C.B.); prlucas@esel.pt (P.L.)

**Keywords:** hospitals, nursing, personnel turnover, systematic review, work environment

## Abstract

Background: Currently, there is a global shortage of nurses, which has a negative impact on health institutions, mainly on the quality of care provided. The quality of nursing care depends on several factors, including the nursing practice environment, which has been stated as a fundamental element for the successful functioning of health systems. Scientific evidence shows that favorable practice environments contribute to nurse retention, and unfavorable environments increase nurses’ turnover intention. Retaining nurses is essential to ensure the sustainability of healthcare organizations and the quality of nurse care. In the current shortage scenario, this issue has become even more significant. Objective: This review aims to identify systematic literature reviews that describe the characteristics of nursing practice environments that contribute to nurse retention or turnover intention in hospitals. Methods: The umbrella review will follow the Joanna Briggs Institute methodology, the Preferred Reporting Items for Systematic Review and Meta-Analysis reporting guidelines, and the Preferred Reporting Items for Overviews of Reviews guidelines. A search with indexed terms will be performed in various databases, and two reviewers will identify, select, and extract studies, while a third reviewer will resolve any disagreements. Results: This protocol provides a structured approach for conducting the review, and the initial searches have identified 440 articles, with no previous protocol or umbrella review identified, underscoring the importance of this research. Conclusions: This study will enhance the dissemination of scientific evidence regarding nursing practice environments, thereby contributing to an improved understanding of factors affecting nurse retention and turnover intention.

## 1. Introduction

In the 21st century, providing quality healthcare has become a priority. To ensure the quality of nursing care, it is crucial to consider all the factors that affect working conditions [1,2]. Among the factors that contribute to the smooth functioning of healthcare systems, a favorable nursing environment is one of the most important [2,3]. The nursing practice environment has a direct impact on the quality of nursing care, patient safety, patient satisfaction, effectiveness of care, and the recruitment and retention of nurses. Studies have shown that improving the nursing practice environment can lead to many positive outcomes for healthcare organizations and patients. The available scientific evidence shows that favorable nursing practice environments are linked with lower rates of negative outcomes, job dissatisfaction, and mortality [3,4,5]. Given these findings, healthcare providers must create and maintain an environment that fosters high-quality nursing practices [2,6].

Nursing practice environments are defined by the organizational characteristics of a work context that either facilitate or constrain the professional nursing practice [7]. The characteristics of the nursing practice environment can be favorable or unfavorable and are associated with variables such as nurse participation in hospital affairs; nursing fundamentals for quality care; nurse manager’s capacity, leadership, and support for nurses; adequacy of staff members and material resources; and good nurse–doctor relations [5]. Favorable nursing practice environments should have positive characteristics and evidence-based nursing practices should be encouraged to promote high-quality care [3,7]. When nursing practice contexts are unfavorable, major concerns can arise, such as increased turnover. Around 3.7 million nurses work in other countries due to unfavorable nursing practice contexts. Currently, there is a global shortage of nurses, and it is predicted that there will be a shortage of 5.7 million nurses worldwide by 2030. Associated with this problem, the migration of nurses increases the disparities in the quality and supply of healthcare across the world [8]. To mitigate the negative effects associated with this issue, governments, employers, and regulators should coordinate actions to promote the existence of a favorable nursing practice environment, contributing to the recruitment, development, retention, and motivation of nurses [8]. A favorable nursing practice environment is crucial for retaining nurses; nurses working in such environments are 28–32% less likely to consider leaving their position [5]. A favorable nursing practice environment significantly increases the three-year retention rate for newly graduated nurses. However, an unfavorable nursing practice environment is correlated with a higher prevalence of burnout, dissatisfaction, and turnover intention, especially for newly graduated nurses [9]. There are currently several scales developed to assess nursing practice environments, such as the nursing work index, which includes several items that make it possible to assess whether the environment is favorable or unfavorable [7].

The COVID-19 pandemic has been a major disruptor in nurse retention and has contributed to increased burnout and risks related to high turnover rates. Pre-pandemic nurse shortages and resource constraints have been exposed and amplified during this globally critical period. The consequences of the pandemic on health professionals affect organizations, making it essential to promote a favorable nursing practice environment to reduce nurse turnover [10]. Organizational turnover in healthcare institutions can have significant effects on nurses, healthcare organizations, and the populations they serve [11]. Turnover refers to the departure of nurses from the organization or the profession and can be either a voluntary or involuntary decision [10]. It is crucial for organizations to recognize that the turnover of nurses can have a significant negative impact on their operations. When a nurse leaves, it can lead to a shortage of essential skills, which requires an interim solution to cover the necessary time to find a substitute. Furthermore, the replacement of a nurse may take some time to reach the same level of skill, leading to a potential break in care continuity, an interruption in the provision of certain services, and a drop in overall productivity. Organizations must understand that retaining their nurses is key to avoiding these potential costs [10,11]. We can analyze the annual costs of employee turnover in different countries. For example, in Australia, it costs USD 48,790; in Canada, USD 26,652; in New Zealand, USD 23,711; and in the United States, USD 20,561 [11]. In these countries, a significant proportion of turnover costs are attributed to replacement, highlighting the importance of retaining nurses. A low level of retention, as indicated by high nurse turnover, can negatively impact organizational costs, productivity, and quality of care. The nursing practice environment plays a crucial role: if the workload is too high and the nursing work environment is unfavorable, this may have a negative impact on retention [11]. Despite the relevance and substantial volume of studies exploring the impact of the nursing practice environment on nurse retention and turnover intention, we identified a significant gap in the literature; there are no umbrella reviews that aggregate and synthesize existing evidence comprehensively and systematically. This protocol aims to respond to an important gap in the literature by providing an integrated, high-quality analysis that consolidates current knowledge on the subject and identifies priority areas for future research. The publication of this protocol is pertinent because it ensures the methodological rigor necessary for the development of an umbrella review, guaranteeing transparency and reproducibility in the research process. Although the topic has already been widely studied, it continues to be of extreme global relevance due to its significant impact on healthcare institutions. This protocol therefore establishes a solid and reliable basis for future reviews, with the objective to identify systematic literature reviews that describe the characteristics of the nursing practice environment that contribute to nurse retention or turnover intention in hospitals. The review is expected to provide an in-depth analysis of the subject, thereby enabling stakeholders to discuss this matter and implement the best practices for nurse retention.

## 2. Materials and Methods

This review protocol was guided by the Preferred Reporting Items for Systematic Reviews and Meta-Analyses (PRISMA-P) [12] guidelines, and to carry out the umbrella review, the Preferred Reporting Items for Overviews of Reviews (PRIOR) guidelines [13], the methodological guidelines of the Joanna Briggs Institute (JBI) [14], and the Preferred Reporting Items for Systematic Review and Meta-Analysis (PRISMA) [15] guidelines will be considered. These guidelines include formulating a clear research question using the PICO format, defining specific inclusion and exclusion criteria for the studies, developing a comprehensive search strategy involving multiple electronic databases, and a systematic study selection process with screening by two independent reviewers and the resolution of disagreements by a third reviewer. In addition, a standardized approach will be used to extract data and assess the methodological quality of the included studies, using tools such as the JBI Critical Appraisal Checklist for Systematic Reviews and Research Synthesis and ROBIS. Data synthesis will be carried out using appropriate methods. The results will be reported according to PRISMA guidelines, ensuring transparency and completeness, and will be interpreted considering the limitations and quality of the studies, highlighting the implications for practice, research, and health policies. 

To formulate the research question, the PICO mnemonic was used (population: nurses who develop their practice in hospital settings; intervention/phenomenon of interest: nursing practice environment; comparison: characteristics of the context/practice environment; outcome: retention or turnover intention). The review question is as follows: What characteristics of the nursing practice environment contribute to the retention, or turnover intention, of nurses in hospitals? The review resulting from the development of this protocol will contribute to the identification of the characteristics of nursing practice environments that can be enforced by nurse managers at different management levels of healthcare organizations, promoting the retention of nurses, mitigating their intention to turnover, and thus contributing to ensuring the continuity and quality of care delivery in hospital settings.

The inclusion criteria are defined as follows:

- Participants: Nurses who have developed their professional activity in hospital settings; nurses with any level of academic training or time of professional experience.
- Phenomenon of interest: All reviews on the characteristics of the nursing practice environment will be considered, and favorable or unfavorable characteristics may be found to be associated with variables such as nurse participation in hospital affairs; nursing fundamentals for quality care; nurse manager’s capacity, leadership, and support for nurses; adequacy of staff and resources; and nurse–doctor collegial relationships [5].
- Results: All reviews that address the nursing practice environment and relate it to the retention or turnover intention of nurses will be considered.
- Context: Hospitals, including public and private entities.
- Types of studies: Systematic reviews of the literature, with or without meta-analysis, systematic intervention reviews, and systematic reviews with mixed methods will be included, with no restrictions on the period of publication of the articles.

Studies will be excluded if they do not explicitly present the research strategy, if they use inappropriate databases, or if they do not carry out a quality assessment of the included studies. Only studies in English, Spanish, and Portuguese will be selected due to the lack of translation methods for other languages. The protocol for this study was registered in the following domain: Protocol 2023110039. doi:10.37766/inplasy2023.11.0039

### 2.1. Research Strategy

The search strategy developed aims to select systematic reviews that have been published on the subject. To map existing scientific evidence, a preliminary search was carried out in the following databases: JBI Database of Systematic Reviews and Implementation Reports, Cochrane Database of Systematic Reviews, Scopus, Web of Science, CINAHL, PROSPERO, INPLASY, Open Science Framework, and Medline. This search also contributed to the definition of the search terms that will be part of the review. The final search will be carried out in the following databases: JBI Database of Systematic Reviews and Implementation Reports, Cochrane Database of Systematic Reviews, CINAHL, Medline, and Scopus. These databases were selected because they include studies in the scientific field of nursing and systematic reviews. The preliminary search took place in one week in December 2023. The search terms and results are described in Table 1.

Table 1 lists 440 articles found through the preliminary search. To conduct the umbrella review, two independent reviewers will review all titles and abstracts based on the predetermined inclusion criteria. If there is disagreement about the inclusion of a study, a third reviewer will be consulted. The reviewers may need to contact the authors for additional information to determine whether to include or exclude a study.

Articles will be compiled from all selected abstracts, and an eligibility check will be performed, based on the inclusion and exclusion criteria, to determine the selected studies. After the articles are selected, an evaluation of their methodological quality will be performed, and the articles obtained will be analyzed. The methodology for selecting the studies will be explained using the PRISMA flow chart, as recommended by the JBI [15]. The search will identify data that answer the research question and objectives, prioritizing articles that relate nursing practice environment characteristics to nurses’ turnover intentions in a hospital context.

The references of the identified studies will be managed using Mendeley Desktop software (Version 1.19.8 © 2008–2020, Mendeley Ltd., London, UK.), and the selection of the included articles will be conducted using the Rayyan software (Qatar Computing Research Institute, Doha, Qatar). 

### 2.2. Evaluation of the Methodological Quality of the Studies

The evaluation of the studies and their methodological quality will be performed by two independent reviewers, using the JBI Critical Appraisal Checklist for Systematic Reviews and Research Synthesis [14]. In cases of disagreement, a third reviewer will be included. If necessary, the reviewers may contact the authors of the studies.

The selected checklist has 11 questions, which should be answered with “yes”, “no” or “uncertain”. The non-applicable option “NA” is also included in the instrument but is rarely applicable. For each question answered affirmatively, 1 point will be awarded, and a predefined score of 7, or higher, will be used to support the decision to include the studies. A score up to 3 will be considered of very low quality, a score between 4 and 6 will be of low quality, a score between 7 and 9 will be of moderate quality, and a score between 10 and 11 will be considered of high quality [14,16].

To avoid bias in the quality assessment, we will use the ROBIS tool for general reviews. The researchers will be given a checklist of ROBIS questions to assess the risk of bias and ensure quality control for each article included in this review [13,17]. The results from the JBI and ROBIS evaluations will play a crucial role in the data synthesis phase. Studies will be categorized based on their quality scores, which will guide the synthesis and interpretation of the findings. Specifically, high-quality studies will provide the primary basis for drawing conclusions. Moderate-quality studies will supplement the findings, offering additional context. Low- and very-low-quality studies will be carefully considered, with their limitations noted and their influence on overall conclusions minimized. By incorporating these tools, we aim to maintain a high standard of methodological rigor and ensure the reliability of our findings. This thorough assessment of methodological quality and risk of bias will enhance the validity of the conclusions drawn from this umbrella review, ultimately contributing to more robust and evidence-based recommendations.

### 2.3. Data Extraction

Data extraction will be performed by two independent reviewers, manually, using the data extraction form recommended by the JBI: the Data Extraction Form for Review for Systematic Reviews and Research Syntheses. According to this form, the following data will be extracted from each study: study details; author/year; objectives; characteristics and number of participants; context; description of the intervention/phenomenon of interest; search details; sources searched; year of included studies; number of included studies; types of included studies; country of origin of included studies; appraisal of instruments used; appraisal rating; methods of analysis; outcomes assessed; results/findings; significance/direction; heterogeneity; comments [14]. A third reviewer may be necessary in cases of a lack of consensus. If data are missing in the studies, the authors may be consulted for additional information.

### 2.4. Data Overview

The data will be synthesized by two reviewers using a table that will summarize information about the different studies, including the phenomenon of interest, methodology, authors, and a summary of the results. A visual indicator will be used, where green will represent factors that favor retention and orange will indicate factors that favor turnover intention. If there is initial disagreement on the interpretation of the data or the assignment of the indicators, the reviewers will have a discussion to reach a consensus. If they cannot reach a consensus, a third senior reviewer will be consulted to make the final decision, ensuring objectivity and adherence to the predefined criteria [14].

## 3. Results

Through the development of this umbrella review, we hope to obtain data on the characteristics of the nursing practice environment that influence the retention or turnover intention of nurses, thus contributing to the dissemination of scientific evidence on the subject. We will use a comprehensive synthesis approach to aggregate the results of the included systematic reviews, ensuring a clear and coherent presentation of how the various characteristics of the nursing practice environment affect nurse retention and turnover intention. This review adheres to the Preferred Reporting Items for Systematic Review and Meta-Analysis Protocols (PRISMA-P) [12] and the Preferred Reporting Items for Overviews of Reviews (PRIOR) guidelines [13]. The PRISMA-P checklist has guided the transparent and systematic development of our review protocol, ensuring that all methodological steps are clearly documented and replicable. The PRIOR checklist will ensure that our synthesis of the systematic reviews is conducted rigorously, covering essential aspects such as the clarity of research questions, the comprehensiveness of search strategies, the rigor of study selection, quality assessment, and transparency in data synthesis.

Our comprehensive search strategy identified 440 articles across multiple databases. The results will be reviewed based on predetermined inclusion criteria, and data from the included studies will be extracted and analyzed. It is hoped that this umbrella review will identify specific characteristics of the nursing practice environment that significantly influence the retention and turnover intentions of nurses in hospital settings. These characteristics include the participation of nurses in hospital affairs, the leadership and support of nursing managers, the adequacy of staff numbers and material resources, and the quality of relationships between nurses and doctors [2,3,4,5,7,18,19,20]. By mapping these characteristics, the review will provide a comprehensive understanding of how different elements of the work environment can affect nurse turnover or retention. In addition, the use of the PRIOR checklist will ensure that the findings are presented in a standardized and reproducible manner, enhancing the reliability of the conclusions. This approach will allow us to identify gaps in the current literature and propose areas for future research. The thorough documentation and adherence to established reporting standards will also facilitate the application of the findings in practical settings, guiding hospital managers and policymakers in implementing effective strategies to improve nurse retention and reduce turnover.

Ultimately, this umbrella review aims to consolidate the existing scientific evidence on the nursing practice environment, providing a valuable resource for improving nurses’ working conditions and enhancing the quality of care provided. Once the review is complete, we will present an in-depth discussion of the findings, exploring in detail the implications of the characteristics identified and offering recommendations based on the consolidated evidence. This process will allow us not only to consolidate the existing evidence on the nursing practice environment, but also to generate a valuable resource for improving the nursing practice environment in hospitals, and consequently improving the quality of nursing care and nurse retention.

## 4. Discussion

The results of this review will have significant implications for nursing policies and practices. In general, identifying the characteristics of the practice environment that favor nurse retention can help hospital managers and policymakers create more supportive work environments, thereby reducing turnover rates. Ultimately, a better practice environment will not only benefit nurses, but also improve the quality of care provided to patients and the efficiency of healthcare organizations. The nursing practice environment is a complex variable that affects many crucial aspects, including the quality of nursing care, job satisfaction, patient safety, the effectiveness of care, organizational efficiency, and the retention and recruitment of nurses [2,3,4,5,7,18,19,20]. Favorable practice environments significantly contribute to retaining professionals, while unfavorable environments increase the intention to leave [9].

Currently, the World Health Organization (WHO) is warning about the shortage of health professionals, especially nurses, highlighting the urgent need for interventions. The International Centre on Nursing Migration emphasizes that the nursing practice environment directly influences nurse retention and turnover intention, which can exacerbate inequalities in the response capacity of health institutions [5]. Unsatisfying working conditions and poor salaries have been identified as conditions that contributes to the migration of nurses, as well as the need to hold down more than one job, therefore adding to the shortage of health professionals in many countries. Making practice environments and pay conditions better will reduce these migratory flows, improve nurse retention, and reinforce the importance of interventions that create more attractive conditions and therefore promote retention [8]. Moreover, turnover represents high costs for healthcare organizations, reinforcing the importance of promoting favorable nursing practice environments [8,11].

The results of this review will not only highlight priority areas for future interventions and research, but will also help target specific efforts to improve the quality of the nursing practice environment. By identifying and mapping these characteristics, hospital managers and policymakers will be better equipped to implement changes that promote nurse satisfaction and retention, thereby improving the efficiency and effectiveness of healthcare organizations.

## 5. Conclusions

The review, resulting from the development of this protocol, will contribute to the overview of the available information on the characteristics of the professional practice environment that influence nurses’ retention or turnover intentions. This study will provide strong contributions for nurse managers and policymakers to intervene in the improvement of healthcare organizations. As implications for research, areas where the development of additional studies is pertinent may be identified. As implications for clinical practice, the contributions of this review could guide the development of measures to promote the retention of nurses in hospital institutions, mitigating the adverse effects associated with high turnover rates. We aim to make an important contribution to the scientific evidence on this subject. Additionally, this review followed the Preferred Reporting Items for Systematic Review and Meta-Analysis Protocols (PRISMA-P) guidelines and the Preferred Reporting Items for Overviews of Reviews (PRIOR) guidelines, ensuring a systematic and transparent approach throughout the review process [13]. This adherence to PRIOR guidelines guarantees the reliability and comprehensiveness of the findings, providing a solid foundation for evidence-based recommendations. The protocol may be limited by the reliance on existing systematic reviews, which could introduce biases from the original studies. Additionally, variations in the definitions and measurements of the nursing practice environment across studies might affect the synthesis of findings.

## Figures and Tables

**Table 1:** Search terms and results.

Database/Results	Search Terms
Cochrane Database of Systematic Reviews (1)	((Workplace OR Working Conditions OR Work environment)) AND ((Practical Nurses OR Nurses OR Nursing Staff)) AND ((Personnel Turnover OR Intention to leave OR retention)) AND hospital AND ((Review OR systematic OR meta-analysis)) AND ((systematic OR meta-analysis)) NOT ((protocol OR Scoping))
JBI (39)	(Review or systematic or meta-analysis).mp. not (scoping or protocol).m_titl. [mp = text, heading word, subject area node word, title] AND ((Practical Nurses or Nurses or Nursing Staff) and (Personnel Turnover or intention to leave or retention) and hospital and (Workplace or Working Conditions or Work environment)).mp. [mp = text, heading word, subject area node word, title] limit to systematic reviews
SCOPUS (45)	((title-abs-key (workplace)or title-abs-key (working and conditions)or title-abs-key (work and environment)))and ((title-abs-key (practical and nurses)or title-abs-key (nurses)or title-abs-key (nursing and staff)))and ((title-abs-key (personnel and turnover)or title (retention)or title-abs-key (intention and to and leave)))and (title-abs-key (hospital)) and ((title (review)or title-abs (systematic)or title-abs (meta-analysis)) and not (title-abs-key (scoping)or title-abs-key (protocol)))
CINAHL (11)	AB (Personnel Turnover or intention to leave or retention) AND AB (Practical Nurses or Nurses or Nursing Staff) AND AB (workplace OR work environment OR working conditions) AND AB hospital AND TI (Review OR systematic OR meta-analysis) AND AB (systematic OR meta-analysis) NOT TI (protocol OR scoping)
MEDLINE (9)	AB (Personnel Turnover or intention to leave or retention) AND AB (Practical Nurses or Nurses or Nursing Staff) AND AB (workplace OR work environment OR working conditions) AND AB hospital AND TI (Review OR systematic OR meta-analysis) AND AB (systematic OR meta-analysis) NOT TI (protocol OR scoping)
PROSPERO (1)	((Workplace or Working Conditions or Work environment) and (Practical Nurses or Nurses or Nursing Staff) and (Personnel Turnover or intention to leave or retention) and Hospital and (review or systematic or meta-analysis) not (scoping or protocol))
INPLASY (1)	((Workplace or Working Conditions or Work environment) and (Practical Nurses or Nurses or Nursing Staff) and (Personnel Turnover or intention to leave or retention) and Hospital)
Web of Science (9)	Personnel Turnover or intention to leave or retention (abstract) AND Practical Nurses or Nurses or Nursing Staff (abstract) AND workplace OR work environment OR working conditions (abstract) AND hospital (abstract) AND Review OR systematic OR meta-analysis (title) AND systematic OR meta-analysis (abstract) NOT protocol OR scoping (title)
Open Science Framework (324)	Work environment AND retention OR turnover intention Provider: OSF Preprints; Subject: Nursing

## Public Involvement Statement

No public or patient contribution.

## Guidelines and Standards Statement

This protocol was developed in accordance with the Preferred Reporting Items for Systematic Review and Meta-Analysis Protocols (PRISMA-P) guidelines.
